# Increased aortic stiffness in adolescents with type 1 diabetes by cardiac magnetic resonance

**DOI:** 10.1186/1532-429X-17-S1-P409

**Published:** 2015-02-03

**Authors:** Uyen Truong, Amy Baumgarter, Greg Coe, Laura Pyle, Sonali Patel, Kristen Nadeau

**Affiliations:** 1 Children's Hospital Colorado, Aurora, CO, USA; 2 University of Colorado, Aurora, CO, USA

## Background

Cardiovascular disease (CVD) is the number one cause of mortality in patients with type 1 diabetes (T1D). There is evidence that maladaptive vascular changes associated with diabetes have already occurred as early as adolescence and that markers of aortic stiffness has been shown to be important predictors of early CVD risk. In this study, we evaluate the central distensibility, a measure of aortic stiffness by MRI, in adolescents with T1D compared to non-diabetic controls.

## Methods

Adolescents age 12-18 years with T1D were enrolled in the EMERALD (Effects of Metformin on Cardiovascular Function in Adolescents With Type 1 Diabetes) study to evaluate changes in cardiovascular function before and after initiation of metformin. Non-diabetic controls, age 7-21 years, were recruited from a separate study. A cross section of the ascending aorta at the level of the right pulmonary artery was evaluated by through-plane phase contrast imaging within a cardiac magnetic resonance imaging study on a 1.5T Siemens Avanto or 3.0T GE HDxt scanner. Magnitude image through the ascending aorta were analyzed for cross-sectional area at maximal and minimal area. Distensibility was calculated as (Area_max_-Area_min_)_/_Area_max_.

## Results

Twenty-three T1D subjects were evaluated prior to the initiation of metformin. The mean disease duration of diabetes was 8.5 years and the mean HbA1c was 8.8%. Twenty-eight non-T1D controls were also recruited. There was no significant difference in body mass index between the two groups. While the control group was younger than the diabetic group, multivariable linear regression accounting for age shows that the aortic distensibility remains significantly lower in the diabetic group (p=0.0471).

## Conclusions

Central vascular stiffness, as reflected by aortic distensibility, is present in adolescents with T1D and may have significant implication for therapeutic targets to decrease lifelong CVD risk.

## Funding

American Diabetes Association 7-11-CD-08

Juvenile Diabetes Research Foundation JDRF 11-2010-343.

**Table 1:** Demographic and distensibility comparison between adolescents with and without type 1 diabetes

	Type 1 Diabetes (N=23)	Controls (N=21)	p-value
Age (years)*	16.3 ± 2.2	12.8 ± 3.3	<0.0001

Female Gender	14/23	12/21	0.9156

Body mass index percentage (median)	83.1 (range 16.4-99.2)	52.4 (1.5-98.9)	0.1178

Aortic Distensibility *	34 ± 11.2	42.3 ± 11.3	0.0471

*Data presented as mean ± standard deviation.

